# Supplementary material for: Volunteer contributions to Wikipedia increased during COVID-19 mobility restrictions
Source: Sci Rep. 2021 Nov 2;11:21505. doi: 10.1038/s41598-021-00789-3 (PMC8563865; doi:10.1038/s41598-021-00789-3)
Supplement: Supplementary file 1 — Supplementary Information. [file 41598_2021_789_MOESM1_ESM.pdf]

Supplementary Information for Volunteer contributions to Wikipedia increased during COVID-19 mobility restrictions

Thorsten Ruprechter[1,*], Manoel Horta Ribeiro[2], Tiago Santos[1], Florian Lemmerich[3], Markus Strohmaier[4,5], Robert West[2], and Denis Helic[1]

[1]Graz University of Technology, 8010 Graz, Austria
[2]EPFL, 1015 Lausanne, Switzerland
[3]University of Passau, 94032 Passau, Germany
[4]RWTH Aachen University, 52062 Aachen, Germany
[5]GESIS – Leibniz Institute for the Social Sciences, 50667 Cologne, Germany
[*]Corresponding author (ruprechter@tugraz.at)

Wikipedia Statistics

This supplementary section summarizes Wikipedia statistics relevant to this study. First, we visualize the total, monthly, weekly, as well as daily edits to Wikipedia in the year before the COVID-19 pandemic began (2019) in Supplementary Table 1. Secondly, Supplementary Table 2 shows statistics describing the yearly growth of Wikipedia between 2015 and 2020 until October of each year. Finally, Supplementary Table 3 lists all Wikipedias we studied, alongside their automatically detected mobility changepoints (Methods).

Supplementary Table 1: **Edits to Wikipedia in 2019.** Overall, Wikipedia language editions were subject to 193 412 million visits and 319.82 million content edits by non-bot users in 2019.[1,2] This translates to about 530 million visits and 876 thousand edits per day.
[1]Total page views, via Wikimedia Statistics (`https://w.wiki/vkf`)
[2]User edits, via Wikimedia Statistics (`https://w.wiki/vkm`)

In 2019	Views (in Millions)	Edits (in Millions)
**Total**	193 412.78	319.82
**Monthly**	16 117.73	26.65
**Weekly**	3 719.48	6.15
**Daily**	529.9	.876

Supplementary Table 2: **Growth between 2019 and 2020 in the English Wikipedia nearly doubles the relative increase between 2015 and 2019.** Edit growth in Wikipedia stagnated in recent years. From 2019 to 2020, edits grew by 8.41% (2.3 million edits), that is nearly double the growth between 2015 and 2019 (4.48%, or about 1.2 million edits).[1] In 2018 and 2019, human edits even declined in comparison to previous years. Therefore, the developments in 2020 mark a clear difference to the downward trend of edits in Wikipedia in the last 5 years.
[1]Edits by anonymous or registered users, via Wikimedia Statistics (`https://w.wiki/u8R`)

Year	Non-Bot Edits (until September 31st)	Difference to Previous Year		Difference to 2019	
		Edits	Percent	Edits	Percent
2015	25 943 729	–	–	−1 163 251	−4.48
2016	27 095 495	1 151 766	4.44	−11 485	−0.04
2017	27 406 797	311 302	1.15	299 817	1.09
2018	27 374 765	−32 032	−0.12	267 785	0.98
2019	27 106 980	−267 785	−0.98	–	–
2020	29 386 750	2 279 770	8.41	2 279 770	7.76

Supplementary Table 3: **Wikipedia language editions.** The 12 Wikipedia language editions relevant to this study, ordered by the total number of edits (bot and non-bot) in 2019, including mobility and normality changepoint dates (Methods).

**Language**	**Changepoints (2020)**		**Wikipedia Version**	
	**Mobility**	**Normality**	**Code**	**Edits in 2019 (Millions)**
English	03/16	05/21	en	40.56
French	03/16	07/02	fr	7.45
German	03/16	07/10	de	7.33
Italian	03/11	06/26	it	5.80
Japanese	03/31	06/14	ja	3.84
Swedish	03/11	06/05	sv	2.73
Dutch	03/16	05/29	nl	1.78
Korean	02/25	04/15	ko	1.61
Serbian	03/16	05/02	sr	1.29
Norwegian	03/11	06/04	no	0.71
Finnish	03/16	05/21	fi	0.65
Danish	03/11	06/05	da	0.31

Number of editors by daily activity level during COVID-19 mobility restrictions

On Wikipedia, human users contribute edits with varying daily intensity. Wikipedia categorizes editors into five groups, according to their daily activity: 1 to 4, 5 to 24, 25 to 99, and more than 99 daily edits. We retrieve the number of registered editors (and their activity level) via the Wikimedia REST API and apply DiD analysis to detect significant changes across the editor population (Methods).

We again remove outliers before performing DiD analysis (see Methods) and visualize the results for daily active editors per activity level in Supplementary Figures 1 (1 to 4 edits), 2 (5 to 24 edits), 3 (25 to 99 edits), and 4 (more than 99 edits) as well as the overall daily active editors (Supplementary Figure 5). The results corroborate our previous newcomer and edit volume findings, as the number of editors increases significantly after mobility changepoints. Our findings signal an increase in contribution across all activity levels for the editor population, particularly in large and medium Wikipedias, while results for small Wikipedias remain consistent with pre-pandemic baselines.

Revert rate during COVID-19 mobility restrictions

The supplementary explanations in this section extend the revert rate analysis carried out in *Discussion*. We plot the rolling seven-day average revert rate (rr) in Supplementary Figure 6a as well as logarithmic effects for δ_{rr} captured by DiD analysis with revert rate as the dependent variable in Supplementary Figure 6b for large (top), medium (middle), and small (bottom) Wikipedias. We find recession of revert rates for most Wikipedias during the initial weeks of mobility restrictions, possibly indicating a reduction in negative contributions that need to be reverted (e.g., vandalism). Coefficient values for δ_{rr} support this sentiment for large and particular medium or small Wikipedias. Nevertheless, it must be mentioned that revert rate can not be interpreted so simply, as specific bots periodically refactor revisions (e.g., monthly, quarterly) or some editor groups conduct article maintenance in coordinated events. Such difficult-to-predict patterns might be especially notable in Wikipedias with a generally lower amount of reverts, which is often the case in smaller Wikipedias. However, even these spontaneous patterns that would normally drive up revert rates appear to be mostly muted during the COVID-crisis.

Revert rates in the large English, German, and French Wikipedias drop after mobility restrictions come into effect in March 2020 (Supplementary Fig. 6a, top). For English, we observe an average revert rate of 0.09 in the month before mobility restrictions take effect and 0.08 in the month after. Revert rate for both German and French averages approximately 0.06 before the changepoints, but reaches respective minima of 0.043 and 0.045 in the subsequent weeks. DiD analysis and corresponding δ_{rr} (Supplementary Fig. 6b, top) confirm significant relative decreases by measuring respective logarithmic effects of -0.327 and -0.329 for the French and German Wikipedia, signaling a 28%-decline for both Wikipedias ($e^{-0.327} \approx e^{-0.329} \approx 72\%$ of previous levels). Italian shows a similar drop in revert rate (-23%). Relative decrease for English is considerably lower (-11%), but is deemed significant

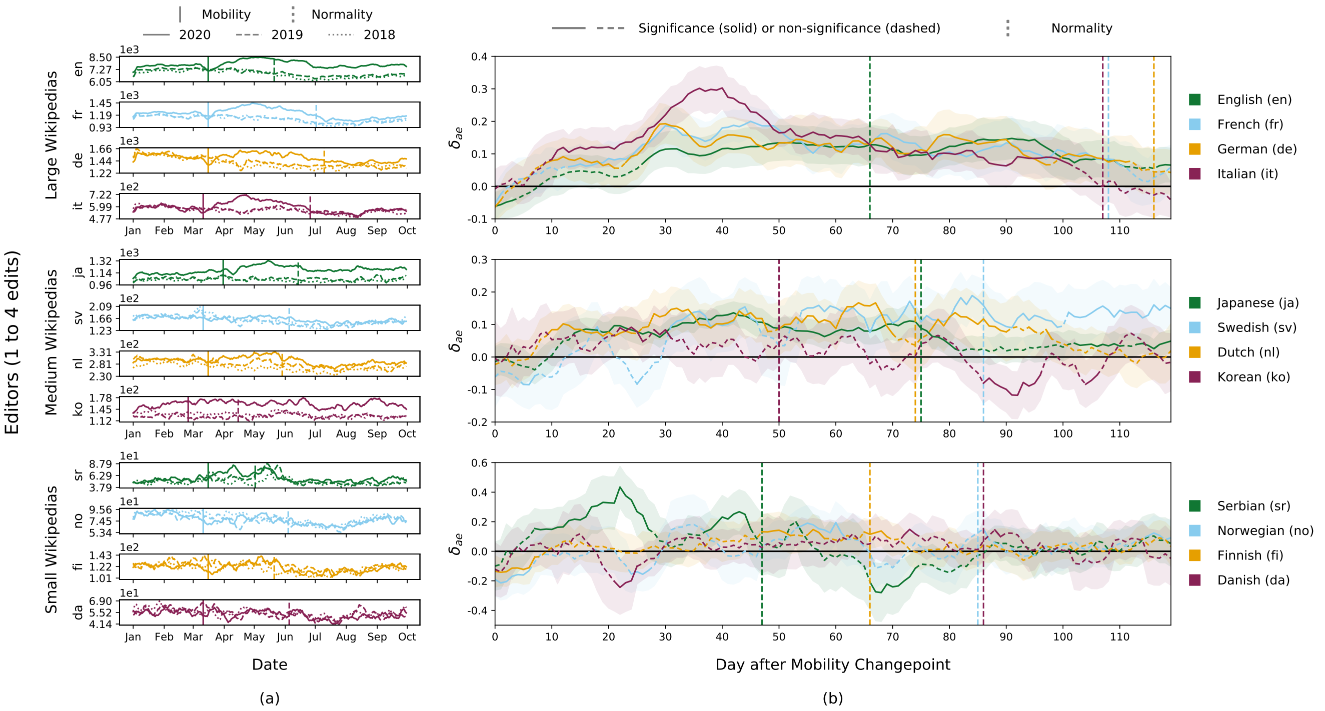

Supplementary Figure 1: **Editors with 1 to 4 edits during COVID-19 mobility restrictions.** **a**, We depict the seven-day rolling average of active registered editors with an activity level of 1 to 4 daily edits in the context of COVID-19 mobility restrictions, delineated via mobility and normality changepoints. **b**, We show the relative change in active registered editors (ae) with 1 to 4 edits per day as retrieved from the DiD via δ_{ae} (95% confidence intervals as two standard deviations). The number of editors with 1 to 4 daily edits increases significantly after mobility restrictions come into effect, for all but a few medium and small Wikipedias.

by our DiD analysis. Altogether, we find significant decreases in revert rate for all large Wikipedias.

Most medium and small Wikipedias seem to not exhibit considerable negative effects for revert rates (Supplementary Fig. 6a, middle and bottom). However, instead of explicitly showing visible dips in the revert rate graphs, the general level seems to be subdued during the investigated periods in 2020, especially close to the mobility changepoints. As an exception, Korean is the only language version that increases its revert rate during times of mobility restrictions, from 0.06 before the changepoint to a maximum of 0.075 in the month thereafter. DiD analysis reveals significant relative decreases in δ_{rr} (Supplementary Fig. 6b, middle and bottom) for the medium Japanese (-42%), Dutch (-28%), and Swedish (-28%) Wikipedias within two months post changepoint, as well as for the smaller Norwegian (-63%), Serbian (-56%), and Danish (-48%) Wikipedias. We explain some of these significant effects by the generally higher revert rate in the same period in previous years. Although our DiD analysis uncovers these significant short-term declines in revert rates for medium and small Wikipedias, results must be taken with a grain of salt due to the aforementioned nature of reverts in smaller Wikipedias.

Edit volume in articles not related to COVID-19

We investigate the impact of articles strongly related to COVID-19 (Methods) on the edit volume on Wikipedia. Supplementary Table 4 lists information about the total percentage of edits to COVID-19 articles, as well as the percentage of edited articles that are related to COVID-19. Supplementary Figure 7 plots the percentage of edits going towards articles strongly related to COVID-19 articles, as well as the overall percentage of edited articles that were strongly related to COVID-19. It appears that edits to COVID-19 articles make up an insignificant account of daily activity in most Wikipedias (mostly $< 4\%$), whereas some Wikipedias, for example German (at one point 15% daily COVID-19 edits), have a somewhat higher but short-lived affinity for COVID-19 topics.

To quantify the effect of COVID-19 articles on overall edit volume, we perform the same DiD analysis for edit volume as in *Results*, this time specifically excluding edits to articles that are strongly related to COVID-19. Supplementary Figure 8 visualizes the performed DiD analysis for edit volume, excluding edits to COVID-19 articles.

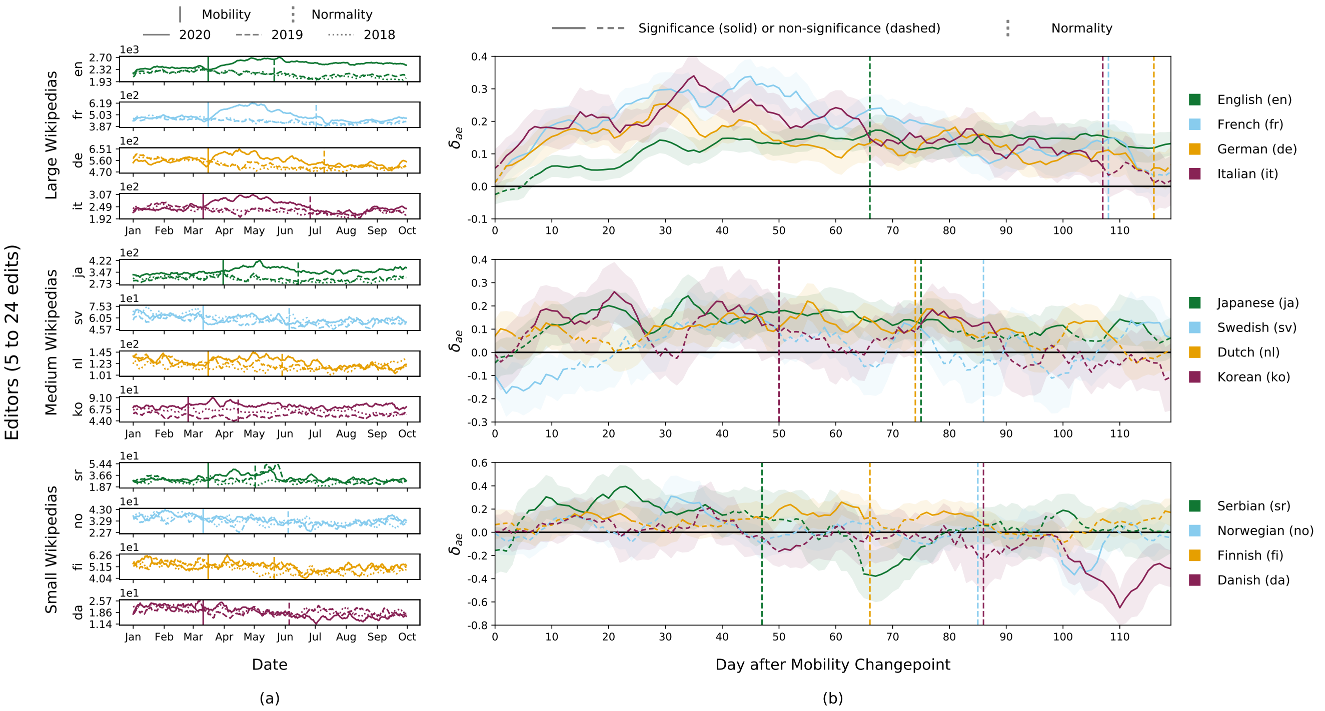

Supplementary Figure 2: **Editors with 5 to 24 edits during COVID-19 mobility restrictions.** **a**, We depict the seven-day rolling average of active registered editors with an activity level of 5 to 24 daily edits in the context of COVID-19 mobility restrictions, delineated via mobility and normality changepoints. **b**, We show the relative change in active registered editors (ae) with 5 to 24 edits per day as retrieved from the DiD via δ_{ae} (95% confidence intervals as two standard deviations). In all large and most medium Wikipedias the number of editors with 5 to 24 daily edits significantly increases over longer periods of time, while smaller Wikipedias do not consistently increase their editor numbers for this activity level.

We find that excluding edits to COVID-19 articles does not significantly alter the results reported beforehand.

Sum of contributed information during COVID-19 mobility restrictions

As a supplementary analysis, we investigate the total amount of contributed information as the sum of changes in bytes during COVID-19 mobility restrictions. For each day and Wikipedia language edition, we accumulate the difference in bytes between all revisions and their parent revisions. We then remove outliers and perform a DiD analysis of the contributed information in bytes.

Although the sum of contributed content in bytes increases in several languages after the imposed mobility changepoints, we find that the surplus in contributed information does not reach the found surplus for edits and newcomers (Supplementary Fig. 9). Moreover, we detect no significant decline in contributed information for all analyzed Wikipedias. The results are similar when excluding edits to COVID-19 articles, but not considering these articles reduces the total amount of contributed information in bytes for some language editions.

Robustness checks for edit volume, newcomers, and revert rate

We perform variations of our DiD experiments for edit volume, newcomers, and revert rate. Supplementary Figures 11, 12, and 13 visualize DiD with a 14-day post-changepoint period. Supplementary Figures 14, 15, and 16 depict DiD with seven-day post-changepoint periods, but move mobility changepoints to seven days before the actual dates. Similarly, Supplementary Figures 17, 18, and 19 move mobility changepoints to seven days after the actual changepoints. This seven-day interval is greater than the difference in days a sensitivity analysis of the mobility changepoints yields (Supplementary Fig. 10). Our DiD robustness checks show that longer post-changepoint periods or modified changepoint dates do not significantly influence results and prove the robustness of our methodology.

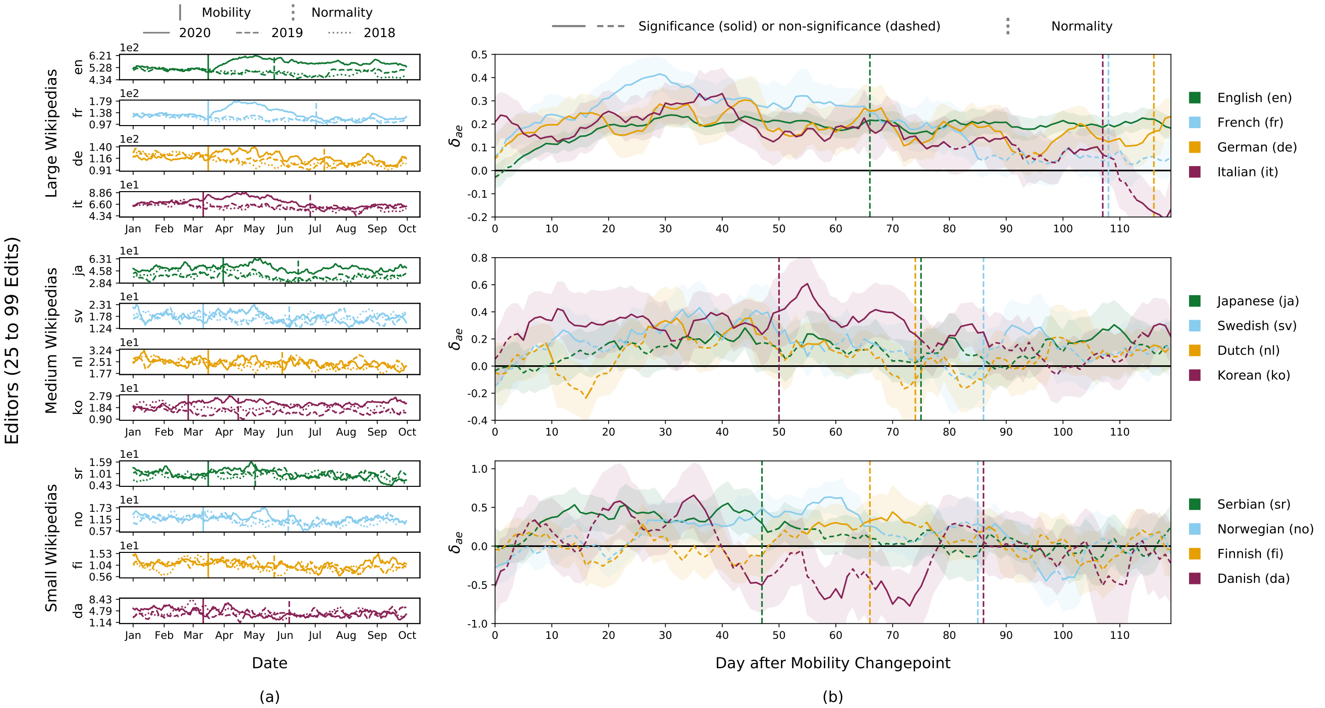

Supplementary Figure 3: **Editors with 25 to 99 edits during COVID-19 mobility restrictions.** **a**, We depict the seven-day rolling average of active registered editors with an activity level of 25 to 99 daily edits in the context of COVID-19 mobility restrictions, delineated via mobility and normality changepoints. **b**, We show the relative change in active registered editors (ae) with 25 to 99 edits per day as retrieved from the DiD via δ_{ae} (95% confidence intervals as two standard deviations). We observe significant increases for the number of registered editors who perform 25 to 99 daily edits during COVID-19 mobility restrictions in large Wikipedias. Results for medium and small Wikipedias are mostly inconsistent.

Supplementary Table 4: **COVID-19 edits and edited COVID-19 articles.** We list the total percentage of non-bot edits to COVID-19 articles as well as the percentage of edited articles that were related to COVID-19 for the total time span between January 1st and September 31st 2020 (second and third column). Furthermore, we show the maxium daily percentage for these two metrics during this period (third and fourth column).

Code	% of Edits to COVID-19 Articles	% of Edited Articles that are COVID-19 Articles	Max. Daily % of Edits to COVID-19 Articles	Max. Daily % of Edited Articles that are COVID-19 Articles
de	2.40	0.44	17.0	1.28
fr	0.73	0.24	3.44	1.00
it	0.29	0.12	1.78	0.72
sr	0.02	0.02	1.27	0.65
no	0.12	0.07	2.19	1.10
ko	0.04	0.03	1.15	0.26
da	0.33	0.16	6.35	1.55
sv	0.25	0.07	3.99	0.49
ja	0.22	0.11	1.49	0.46
nl	0.64	0.21	4.23	1.49
fi	0.75	0.37	4.12	1.43
en	1.03	0.33	4.44	0.82

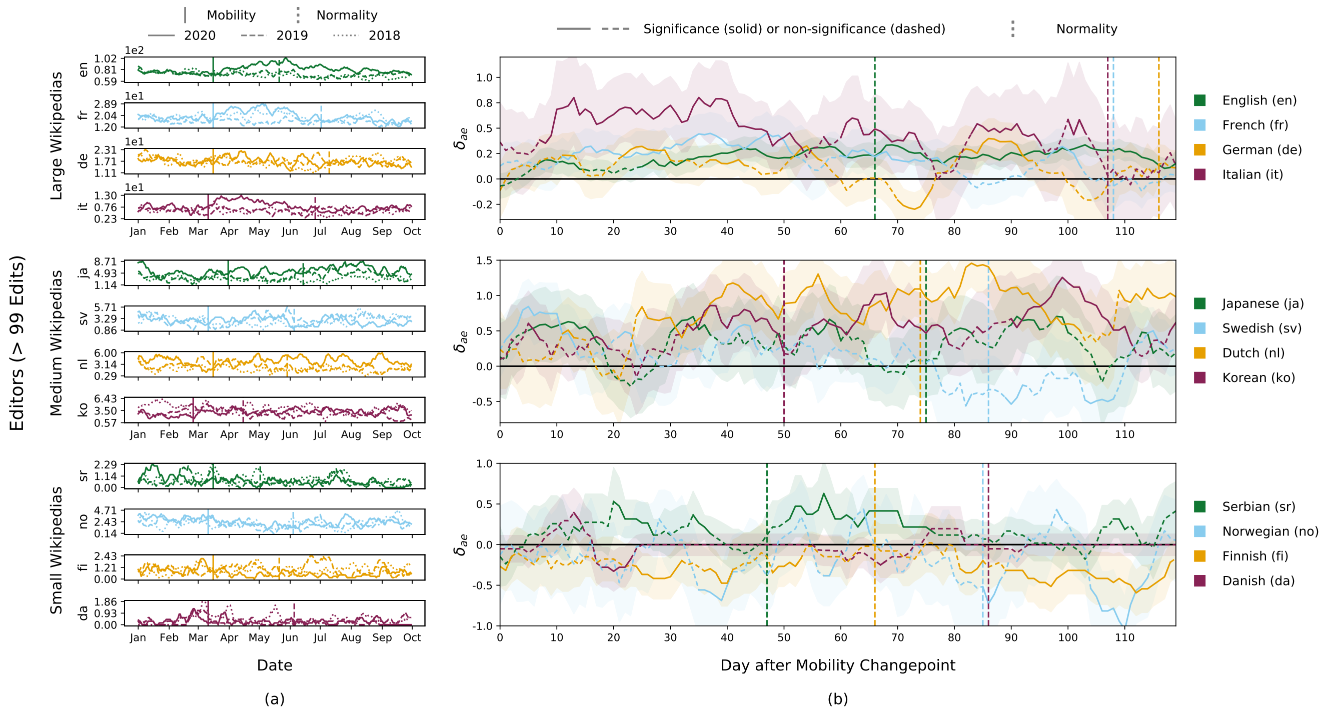

Supplementary Figure 4: **Editors with more than 99 edits during COVID-19 mobility restrictions. a**, We depict the seven-day rolling average of active registered editors with an activity level of more than 99 edits in the context of COVID-19 mobility restrictions, delineated via mobility and normality changepoints. **b**, We show the relative change in active registered editors (ae) with more than 99 edits per day as retrieved from the DiD via δ_{ae} (95% confidence intervals as two standard deviations). For all large Wikipedias, besides German, we find increased counts for editors with more than 99 edits per day. Additionally, some medium Wikipedias exhibit significantly more of these high-intensity editors, while smaller Wikipedias show no strong significant trends.

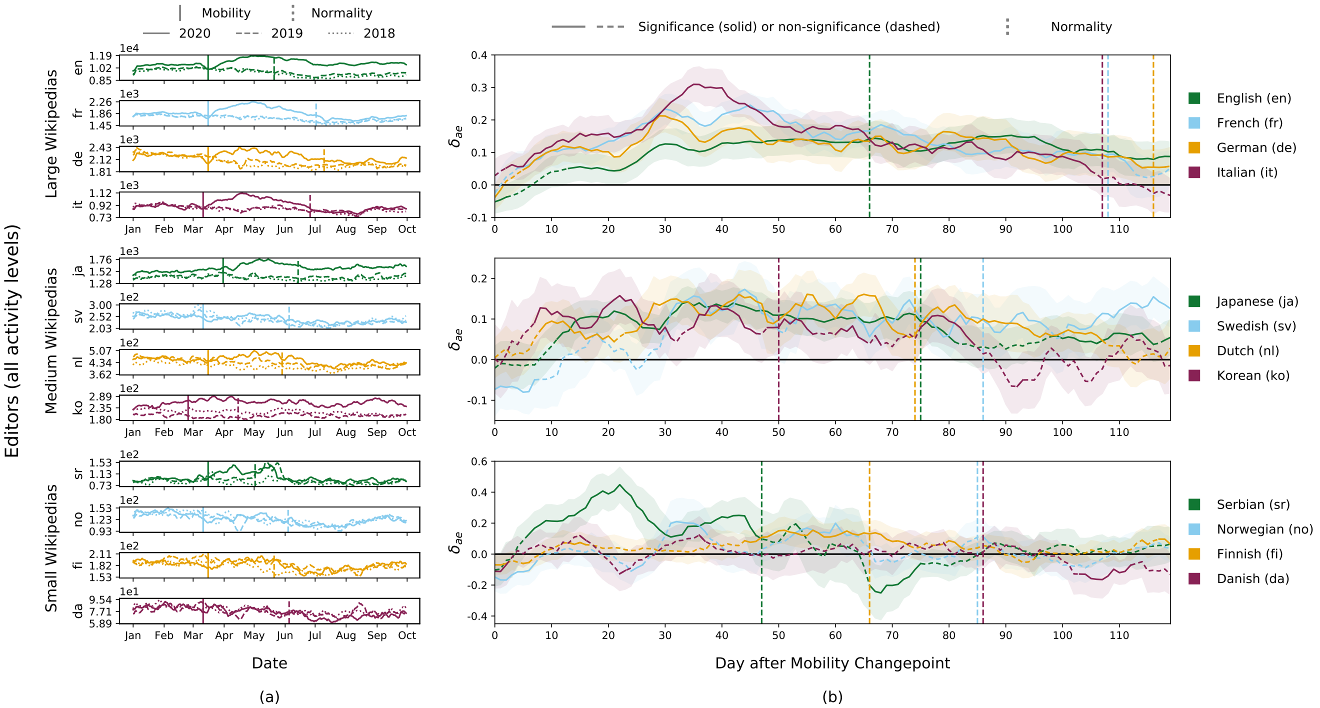

Supplementary Figure 5: **Active editors during COVID-19 mobility restrictions.** **a**, We depict the seven-day rolling average of active registered editors in the context of COVID-19 mobility restrictions, delineated via mobility and normality changepoints. **b**, We show the relative change in active registered editors (ae) as retrieved from the DiD via δ_{ae} (95% confidence intervals as two standard deviations). The number of active editors increases significantly after mobility restrictions come into effect, for all but a few small Wikipedias.

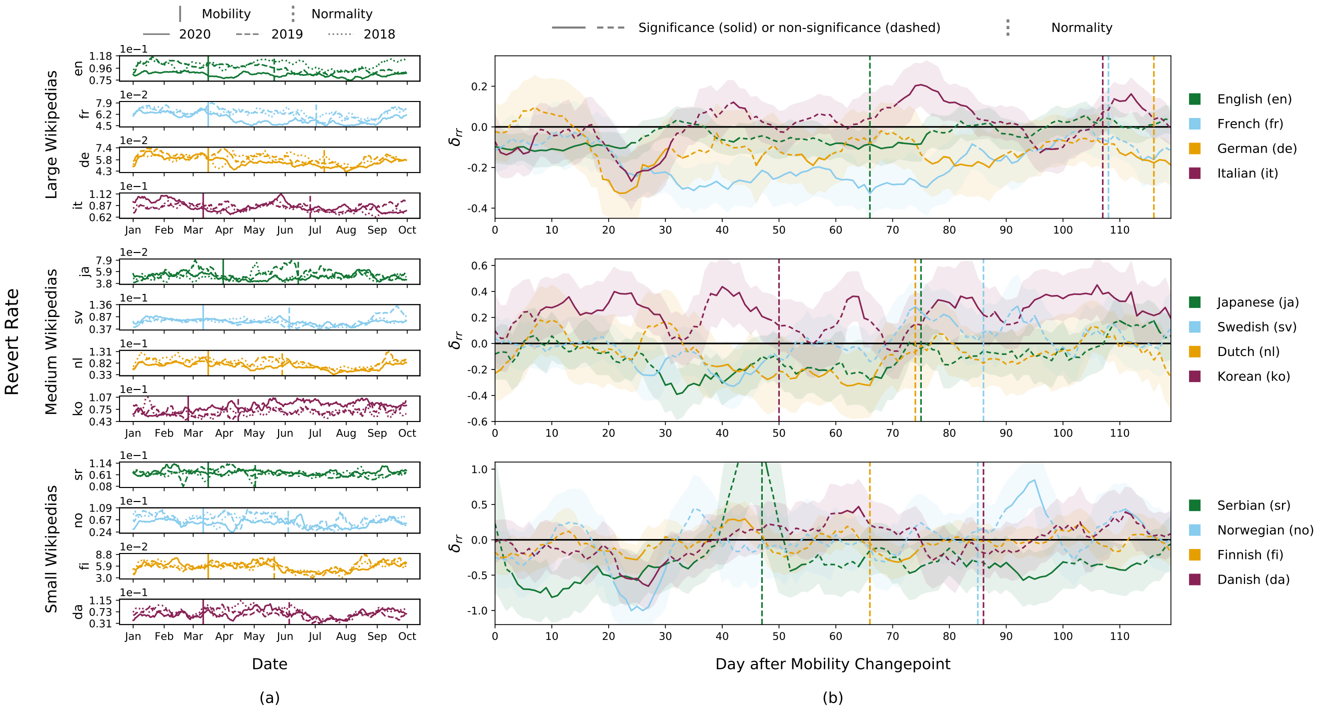

Supplementary Figure 6: **Revert rate during COVID-19 mobility restrictions.** We depict results for revert rate in large (top), medium (middle), and small (bottom) Wikipedias during COVID-19 mobility restrictions, which in the figure we delineate using mobility (when restrictions become effective) and normality (when restrictions are lifted) changepoints. **a**, We plot rolling seven-day average revert rate in 2018, 2019, and 2020 up until October. Even though our analysis showed that newcomers and edit volume increased in 2020, especially after the mobility changepoint, we do not observe increases for revert rate in any Wikipedias. **b**, We calculate the relative change in revert rate (rr) to pre-changepoint periods from the DiD via δ_{rr} (95% confidence intervals as two standard deviations), and plot δ_{rr} for 120 left-aligned seven-day windows (see Methods), beginning with the respective mobility changepoint. We detect no significant increase after mobility restrictions come into effect in virtually all Wikipedias, with the exception of Korean, and even find decreased revert rates for most large and medium Wikipedias.

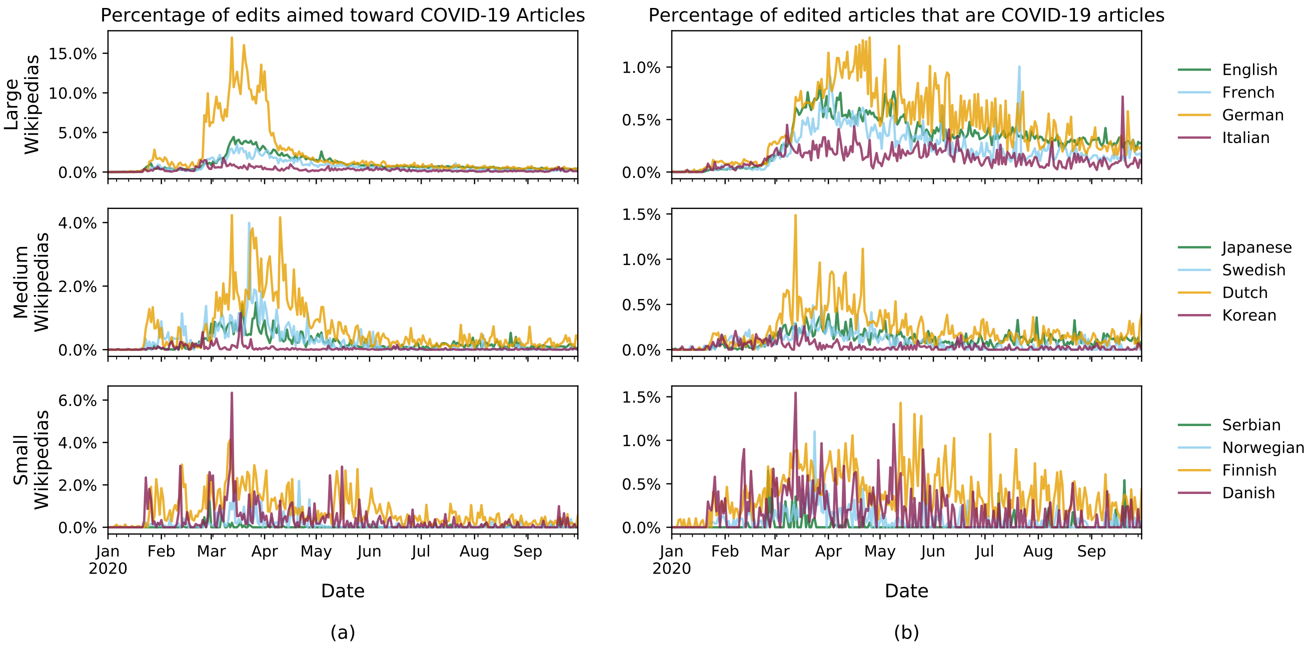

Supplementary Figure 7: **COVID-19 edits and edited COVID-19 articles.** We visualize the percentage of COVID-19 article edits per day and daily edited COVID-19 articles for large (top), medium (middle), and small (bottom) Wikipedias. **a**, We show the daily percentage of non-bot edits to COVID-19 articles between January 1st and September 31st 2020. **b**, We visualize the daily percentage of edited articles that were related to COVID-19 in 2020 until October.

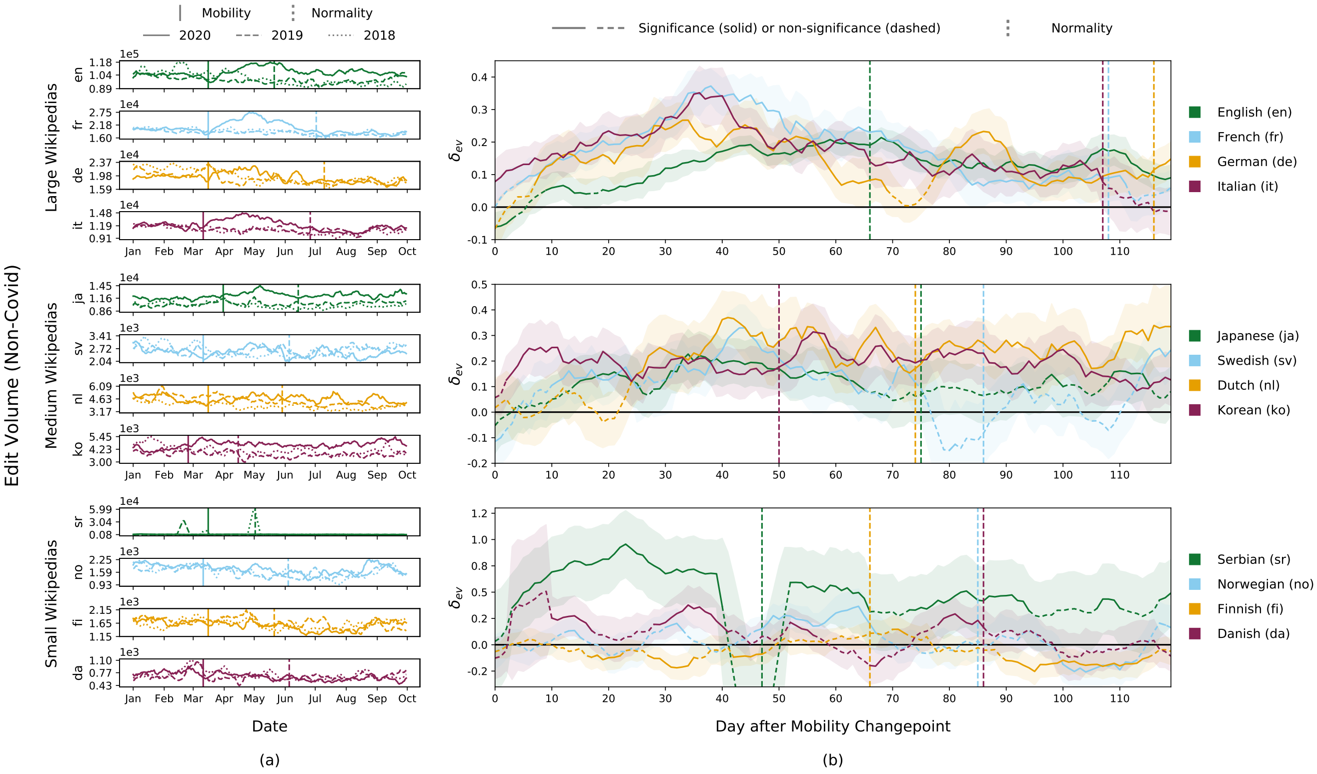

Supplementary Figure 8: **Edit volume in articles not related to COVID-19 during mobility restrictions.** We show edit volume in non-COVID-19 articles in large (top), medium (middle), and small (bottom) Wikipedias during the mobility restrictions, which we delineate using mobility (when restrictions become effective) and normality (when restrictions are lifted) changepoints. **a**, We show rolling seven-day average edit volume generated by human editors for 2018, 2019, and 2020 until October. **b**, We depict relative change in edit volume (ev) as retrieved from DiD via δ_{ev} (95% confidence interval as two standard deviations) and plot δ_{ev} for 120 left-aligned seven-day windows. Edits to articles closely related to COVID-19 mostly only make up a small fraction of daily edits (Supplementary Table 4 and Supplementary Fig. 7). Accordingly, we observe that findings for edit volume excluding COVID-19 edits barely differ from those for overall edit volume depicted in Figure 2. Although a minimal visual effect is observable for a few select Wikipedias (e.g., German), it does not affect significance of our results.

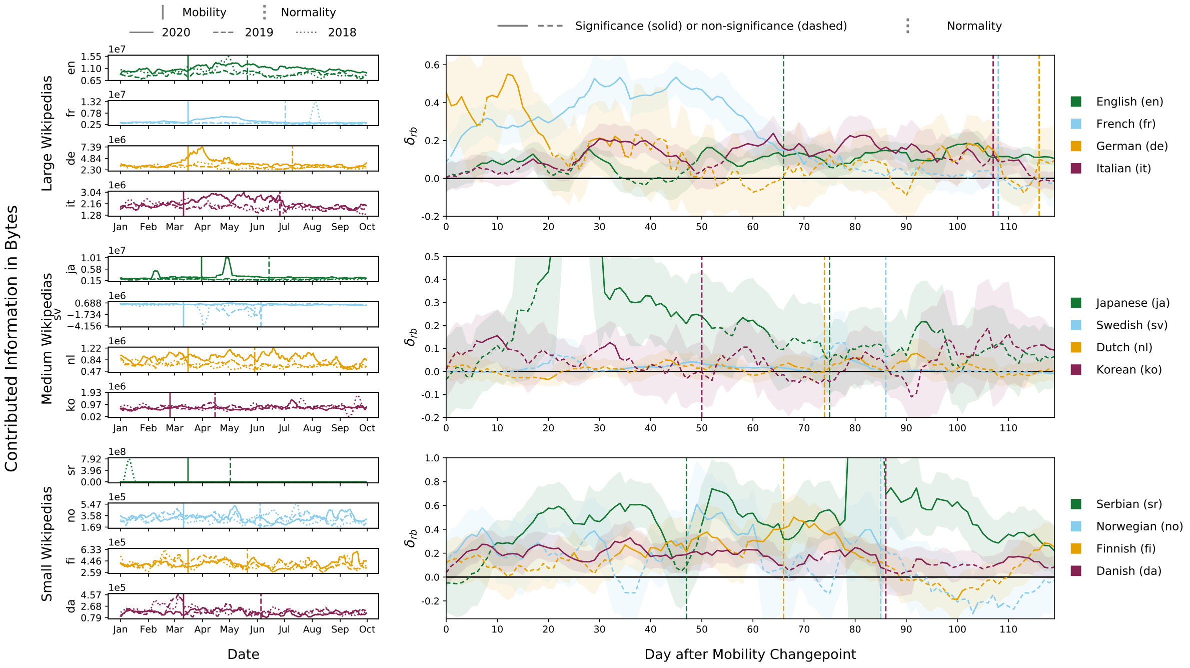

(a) (b)

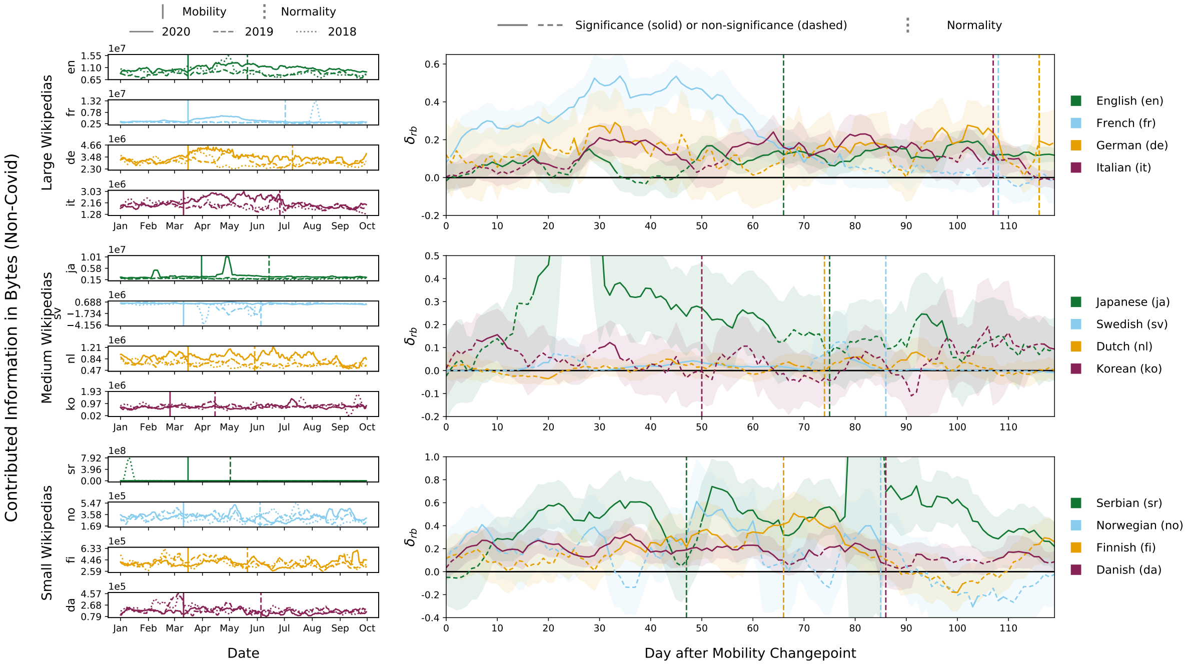

(a) (b)

Supplementary Figure 9: **Total sum of contributed information in bytes during COVID-19 mobility restrictions.** We show the amount of contributed information for all articles (top) as well as only articles not related to COVID-19 (bottom) in the context of mobility and normality changepoints. **a**, We plot the rolling seven-day average daily sum of the difference in bytes for all revisions to their parent revisions.[1] **b**, We visualize the relative change in contributed content in revision bytes (rb) as computed via DiD as δ_{rb} (95% confidence interval as two standard deviations) for 120 left-aligned seven-day windows. Our results depict that contributions do not significantly decline after mobility restrictions take effect, and large as well as some medium and small Wikipedias show increases. However, for most languages the increase is weaker than what was found for newcomers or editors. In general, findings for all articles are not substantially different from those for non-COVID-19 articles.

[1] For some Wikipedias, there are irregularities in the data. Japanese experienced a brief copy-paste vandalism spree in April 2020, where a small number of pages was created and deleted in rapid fashion, with only very few performed edits. In January 2018, multiple large list articles were created in the Serbian Wikipedia. For French, there was a very active period in the beginning of August 2018.

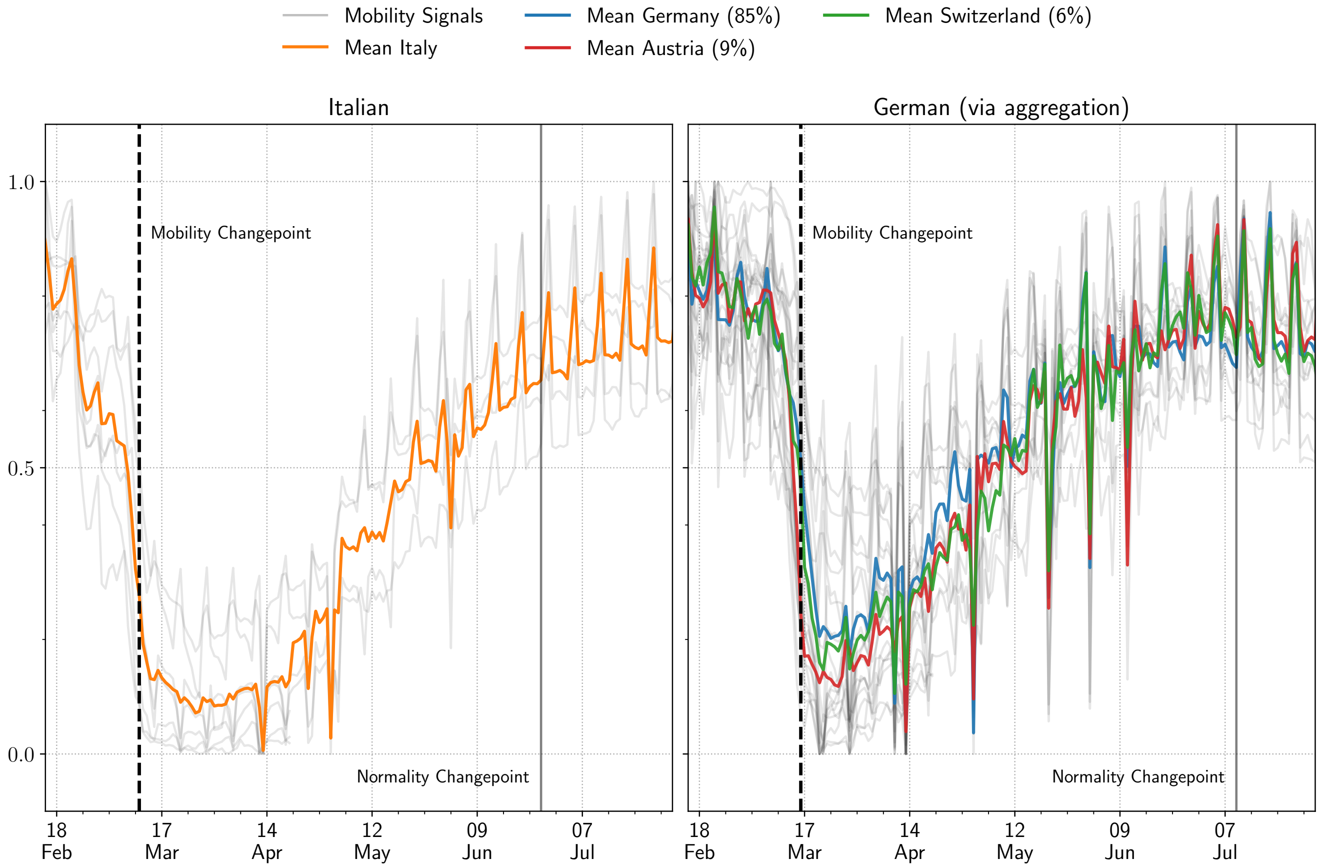

Supplementary Figure 10: **Detecting mobility changepoints.** We depict the mobility and normality changepoints for the Italian and German Wikipedia as obtained from smartphone mobility data. We adopt the changepoint detection approach and this figure from previous work by Horta Ribeiro et al. [1]. Note that for Italian (left) we use data only from Italy, while for German (right), we aggregate data from the three countries that have German as their official language. To aggregate multiple countries for languages such as German, English, and French, we average the mobility changepoints of countries that have these languages as official (or *de facto*) languages[1] and weight the contribution of countries with several official languages (e.g., Switzerland and Canada) by the proportion of native speakers of these languages in each country.[2] We perform a sensitivity analysis of this weighting process, finding that calculating a simple (non-weighted) average for countries speaking multiple languages yields a maximum difference in the changepoint date of 5 days, smaller than the seven-day window we experimented with in our robustness experiments.

[1] See: `https://en.wikipedia.org/wiki/List_of_official_languages_by_country_and_territory`

[2] **German**: Germany 85%, Austria 9%, Switzerland 6%; **English**: US 69%, U.K. 16%, Canada 6%, Australia 5.4%, South Africa 1.5%, Ireland 1.2%, New Zealand 1.1%; **French**: France 63%, Switzerland 1.7%, Canada 10.1%, Cameroon 9.2%, Belgium 8%, Senegal 4.4%, Côte d'Ivoire 3.8%.

References

[1] Horta Ribeiro, M., Gligorić, K., Peyrard, M., Lemmerich, F., Strohmaier, M., and West, R. Sudden attention shifts on wikipedia during the covid-19 crisis. *Proceedings of the International AAAI Conference on Web and Social Media 15*, 1 (May 2021), 208–219.

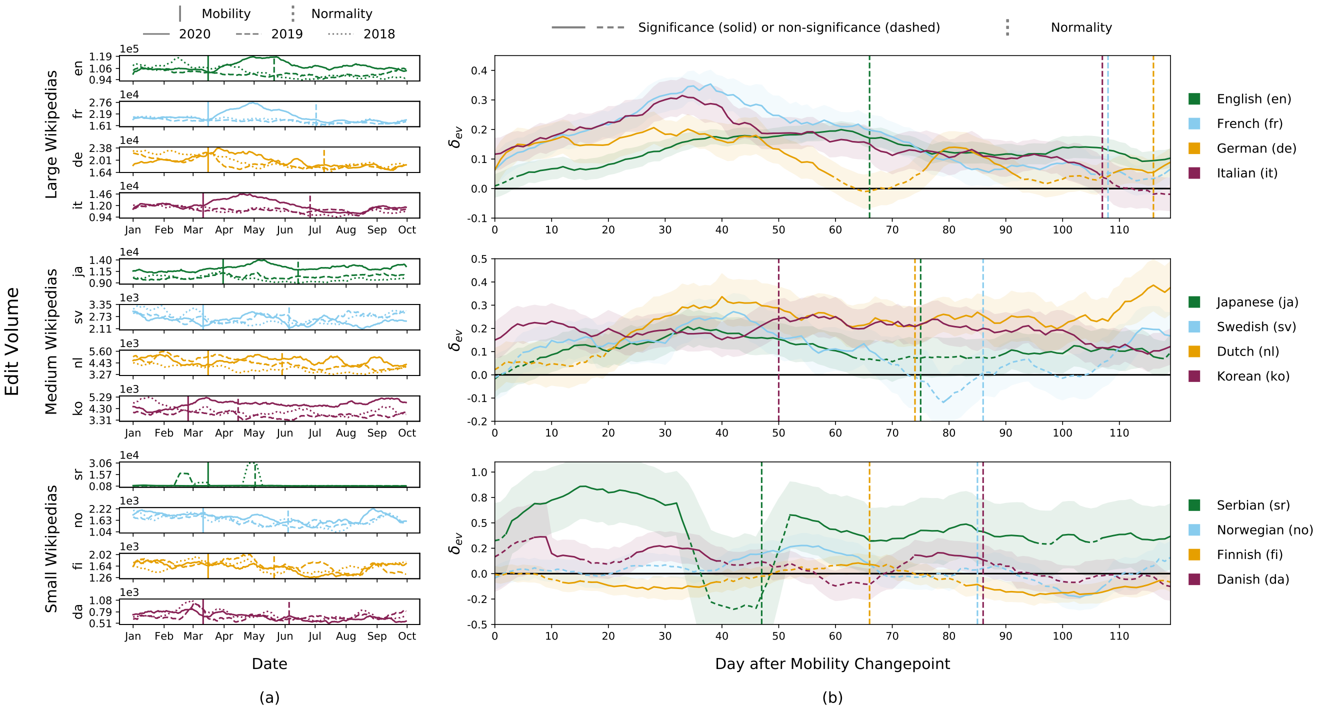

Supplementary Figure 11: **Edit volume during COVID-19 mobility restrictions (14-day windows).** We show results of our 14-day window robustness experiment for edit volume in large (top), medium (middle), and small (bottom) Wikipedias during COVID-19 mobility restrictions, delineated using mobility (when restrictions become effective) and normality (when restrictions are lifted) changepoints. **a**, We show rolling 14-day average daily edit volume generated by human editors for 2018, 2019, and 2020 until October. **b**, We depict relative change in edit volume (ev) as retrieved from DiD via δ_{ev} (95% confidence interval as two standard deviations) and plot δ_{ev} for 120 left-aligned fourteen-day-windows. Edit volume results for 14-day windows represent the same trends and similar significant effects as previous experiments (Figure 2), only smoothening the seven-day window results more.

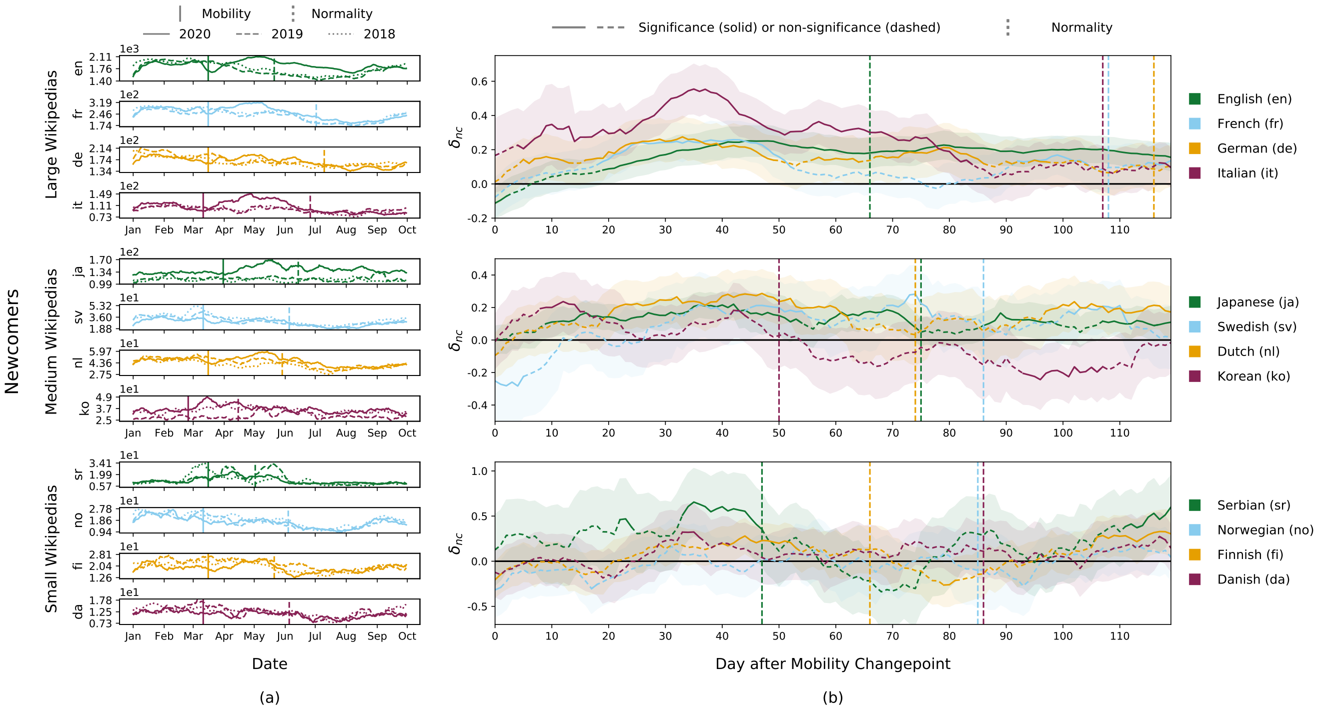

Supplementary Figure 12: **Newcomers during COVID-19 mobility restrictions (14-day windows).** We show results of our 14-day window robustness experiment for newcomers in large (top), medium (middle), and small (bottom) Wikipedias during COVID-19 mobility restrictions, delineated using mobility (when restrictions become effective) and normality (when restrictions are lifted) changepoints. **a**, We show rolling 14-day average newcomer counts for 2018, 2019, and 2020 until October. **b**, We depict relative change in newcomers (nc) as retrieved from DiD via δ_{nc} (95% confidence interval as two standard deviations) and plot δ_{nc} for 120 left-aligned fourteen-day-windows. Newcomer results for 14-day windows represent the same trends and similar significant effects as previous experiments (Figure 3), only smoothening the seven-day window results more.

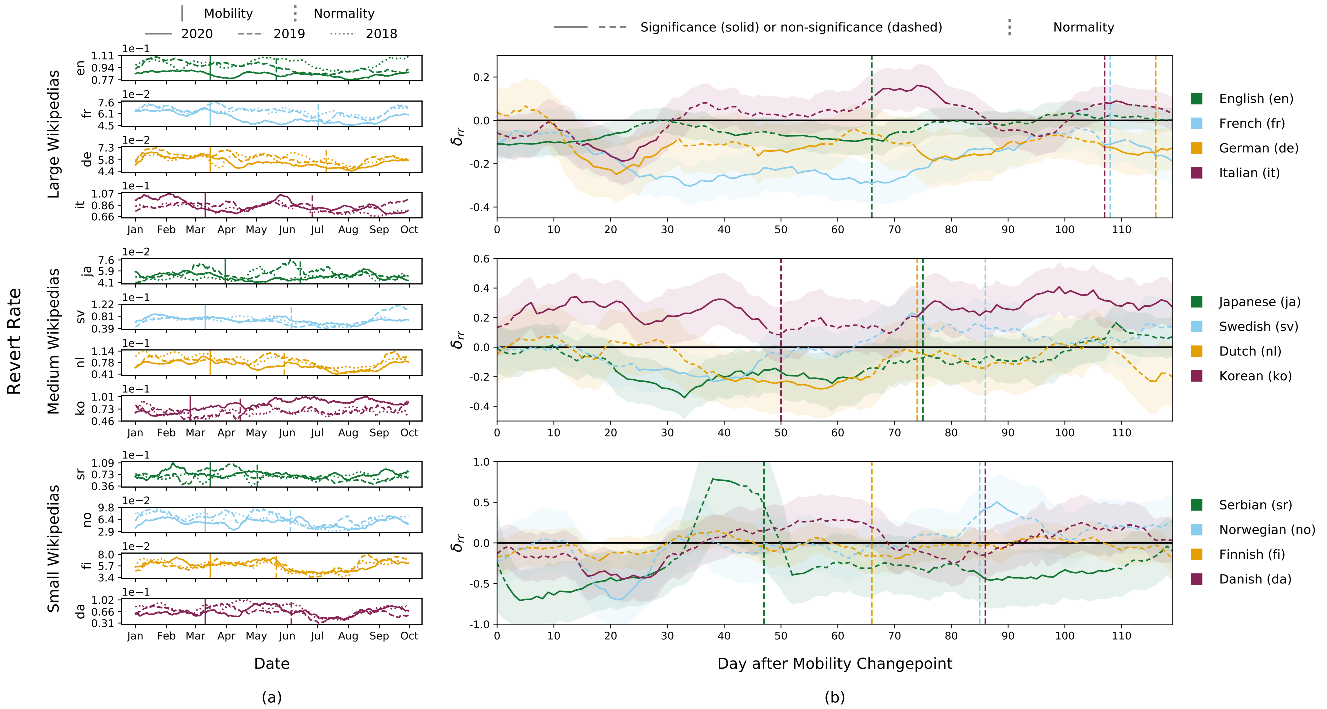

Supplementary Figure 13: **Revert rate during COVID-19 mobility restrictions (14-day windows).** We show results of our 14-day window robustness experiment for revert rate in large (top), medium (middle), and small (bottom) Wikipedias during COVID-19 mobility restrictions, delineated using mobility (when restrictions become effective) and normality (when restrictions are lifted) changepoints. **a**, We show rolling 14-day average revert rate for 2018, 2019, and 2020 until October. **b**, We depict relative change in revert rate (rr) as retrieved from DiD via δ_{rr} (95% confidence interval as two standard deviations) and plot δ_{rr} for 120 left-aligned fourteen-day-windows. Revert rate results for 14-day windows represent the same trends and similar significant effects as previous experiments (Supplementary Fig. 6), only smoothening the seven-day window results more.

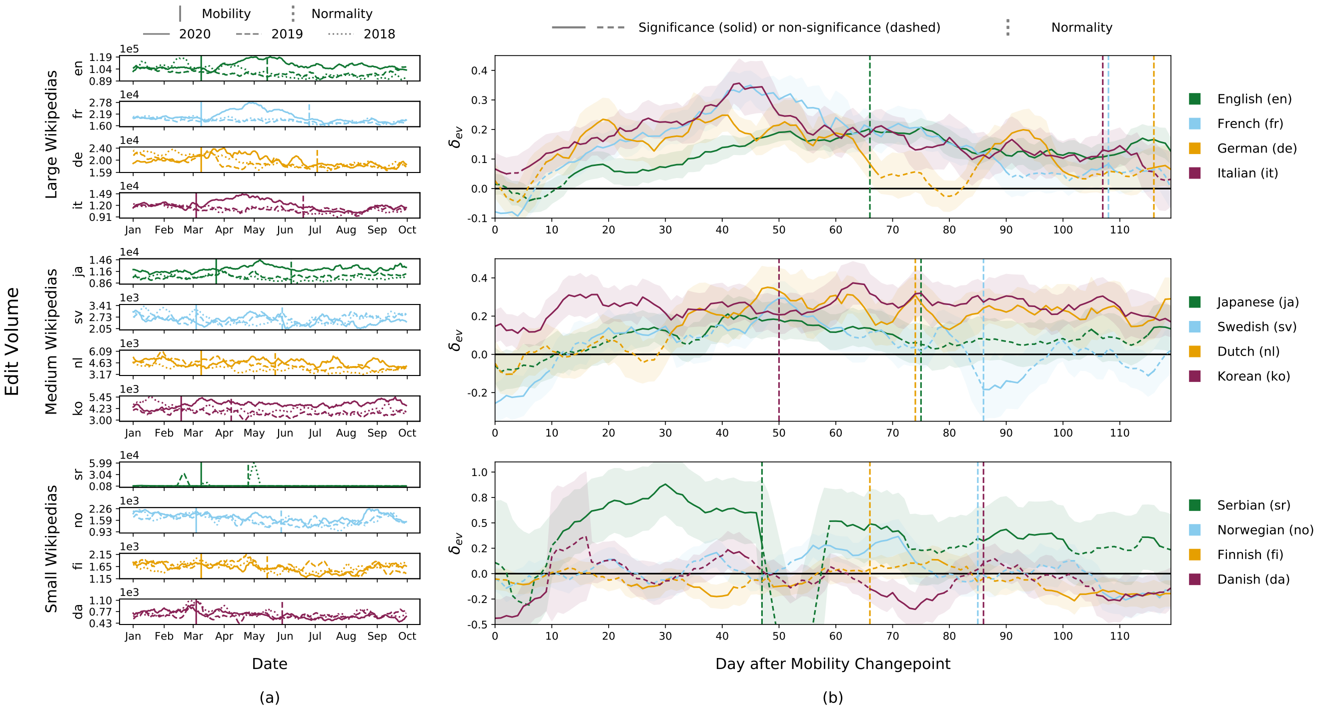

Supplementary Figure 14: **Edit volume during COVID-19 mobility restrictions (beginning 7 days earlier).** We vary our DiD for edit volume by changing the mobility (when restrictions take effect) and normality changepoint (when restrictions are lifted) to 7 days earlier. **a**, We show rolling seven-day average edit volume for 2018, 2019, and 2020 until October. **b**, We depict relative change in edit volume (ev) as retrieved from DiD via δ_{ev} (95% confidence interval as two standard deviations) and plot δ_{ev} for 120 left-aligned seven-day windows. As mobility changepoints generally mark dates of decreased activity, moving the changepoint before these declines lead to the first few days representing a more negative trend and values for later days are slightly lower than in the original experiment (Figure 2), which is an expected effect.

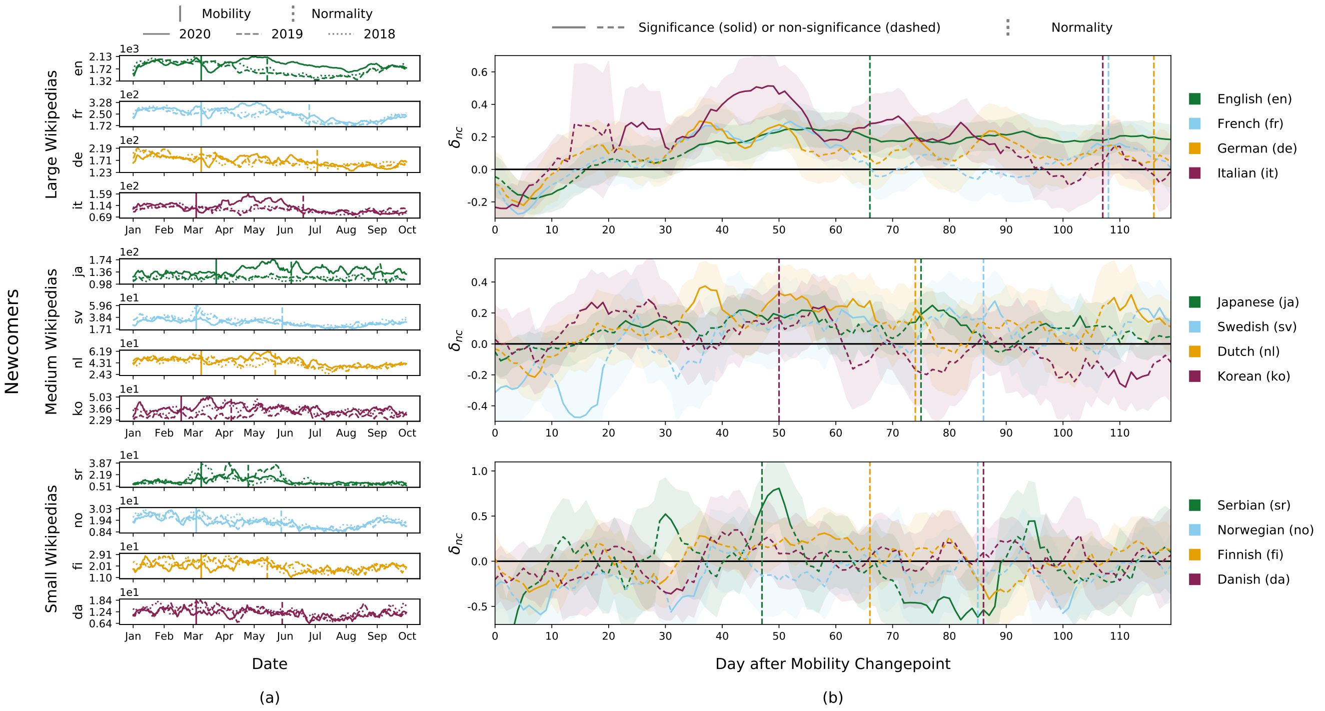

Supplementary Figure 15: **Newcomers during COVID-19 mobility restrictions (beginning 7 days earlier).** We vary our DiD for newcomers by changing the mobility (when restrictions take effect) and normality changepoint (when restrictions are lifted) to 7 days earlier. **a**, We show rolling seven-day average newcomers for 2018, 2019, and 2020 until October. **b**, We depict relative change in newcomers (nc) as retrieved from DiD via δ_{nc} (95% confidence interval as two standard deviations) and plot δ_{nc} for 120 left-aligned seven-day windows. As mobility changepoints generally mark dates of decreased activity, moving the changepoint before these declines lead to the first few days representing a more negative trend, before than recovering and increasing to slightly lower values than in the original experiment (Figure 3), which is an expected effect.

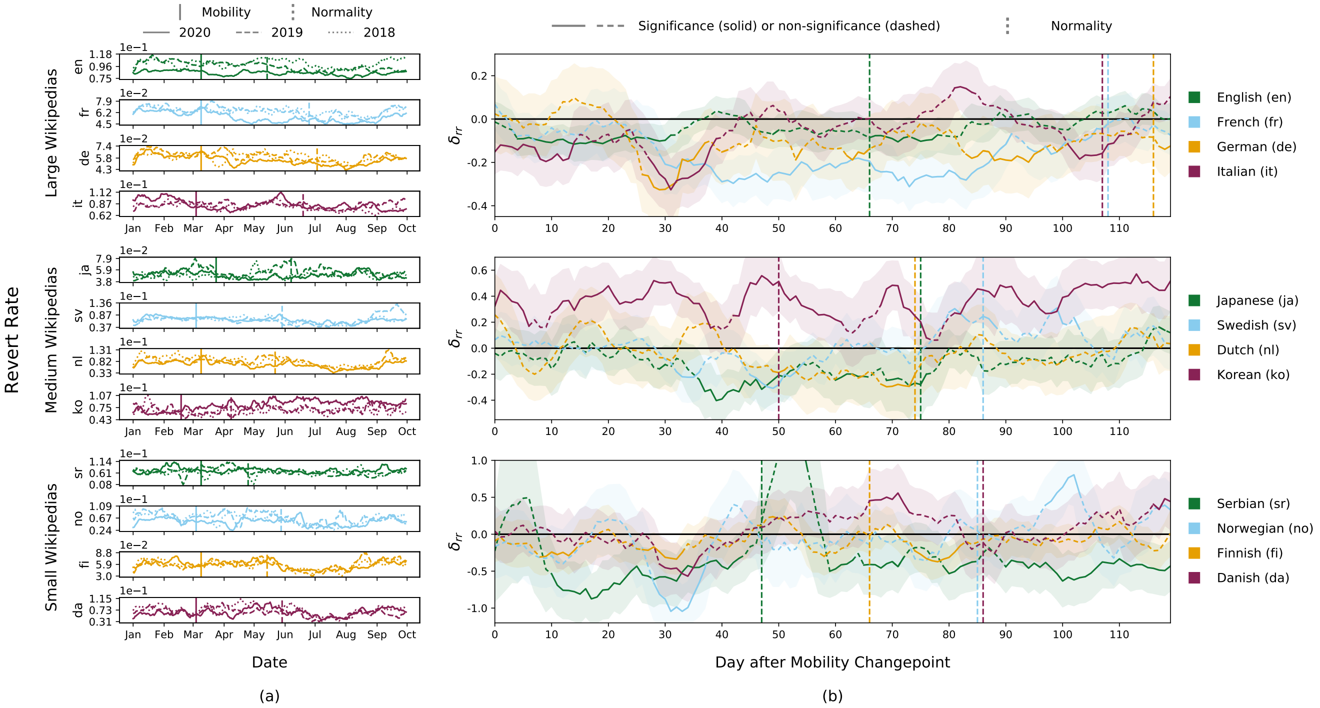

Supplementary Figure 16: **Revert rate during COVID-19 mobility restrictions (beginning 7 days earlier).** We vary our DiD for revert rate by changing the mobility (when restrictions take effect) and normality changepoint (when restrictions are lifted) to 7 days earlier. **a**, We show rolling seven-day average revert rate for 2018, 2019, and 2020 until October. **b**, We depict relative change in revert rate (rr) as retrieved from DiD via δ_{rr} (95% confidence interval as two standard deviations) and plot δ_{rr} for 120 left-aligned seven-day windows. We find no strong differences to the original experiment (Supplementary Fig. 6), as revert rates are relatively stable close to the mobility changepoint.

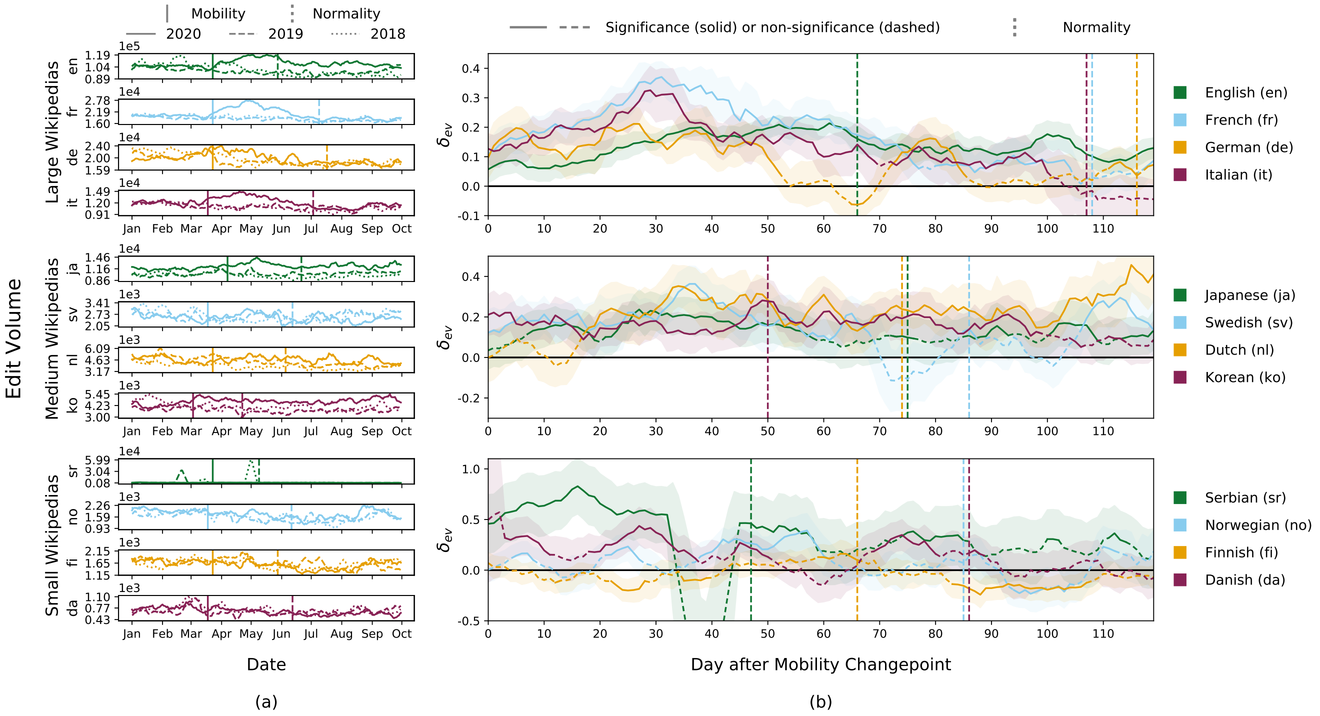

Supplementary Figure 17: **Edit volume during COVID-19 mobility restrictions (beginning 7 days later).** We vary our DiD for edit volume by changing the mobility (when restrictions take effect) and normality changepoint (when restrictions are lifted) to 7 days later. **a**, We show rolling seven-day average edit volume for 2018, 2019, and 2020 until October. **b**, We depict relative change in edit volume (ev) as retrieved from DiD via δ_{ev} (95% confidence interval as two standard deviations) and plot δ_{ev} for 120 left-aligned seven-day windows. As mobility changepoints generally mark dates of decreased activity, moving the changepoint past these declines leads to these lower values now being counted towards the 30-day baseline period, generating overall higher post-changepoint values than in the original experiment (Figure 2).

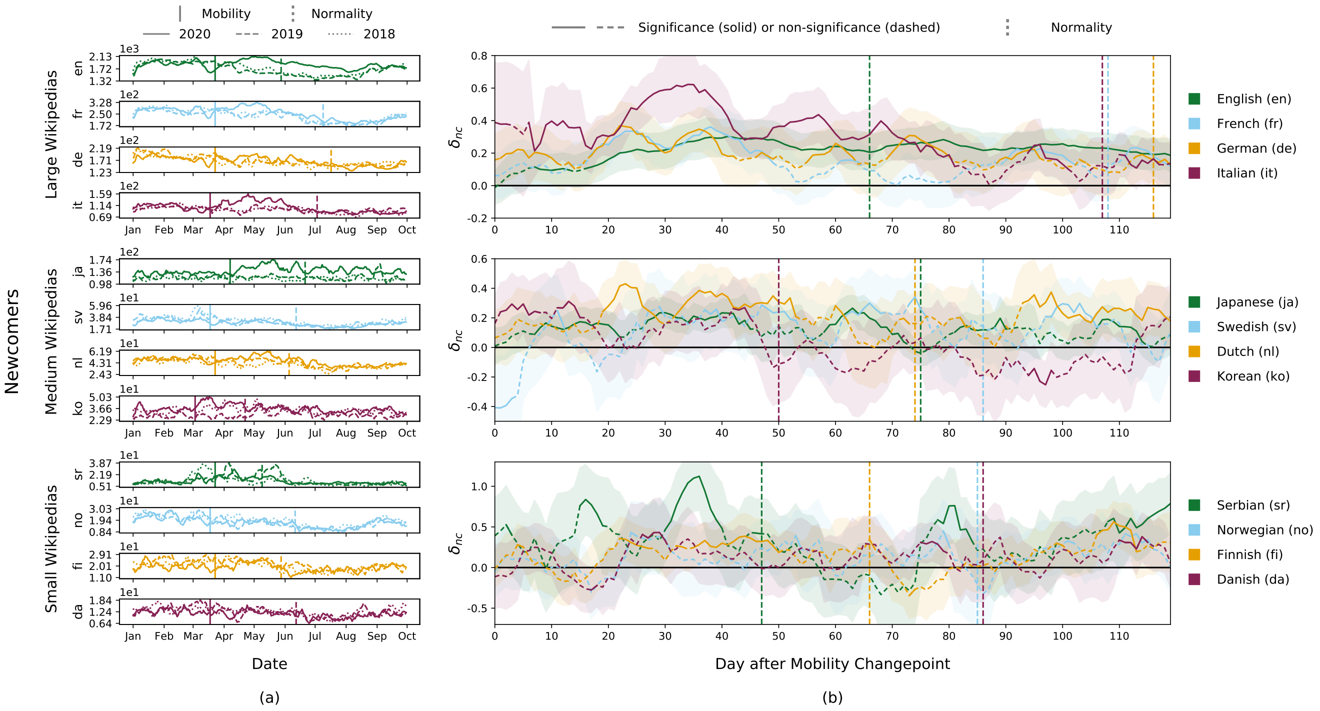

Supplementary Figure 18: **Newcomers during COVID-19 mobility restrictions (beginning 7 days later).** We vary our DiD for newcomers by changing the mobility (when restrictions take effect) and normality changepoint (when restrictions are lifted) to 7 days later. **a**, We show rolling seven-day average newcomers for 2018, 2019, and 2020 until October. **b**, We depict relative change in newcomers (nc) as retrieved from DiD via δ_{nc} (95% confidence interval as two standard deviations) and plot δ_{nc} for 120 left-aligned seven-day windows. As mobility changepoints generally mark dates of decreased activity, moving the changepoint past these declines leads to these lower values now being counted towards the 30-day baseline period, generating overall higher post-changepoint values than in the original experiment (Figure 3).

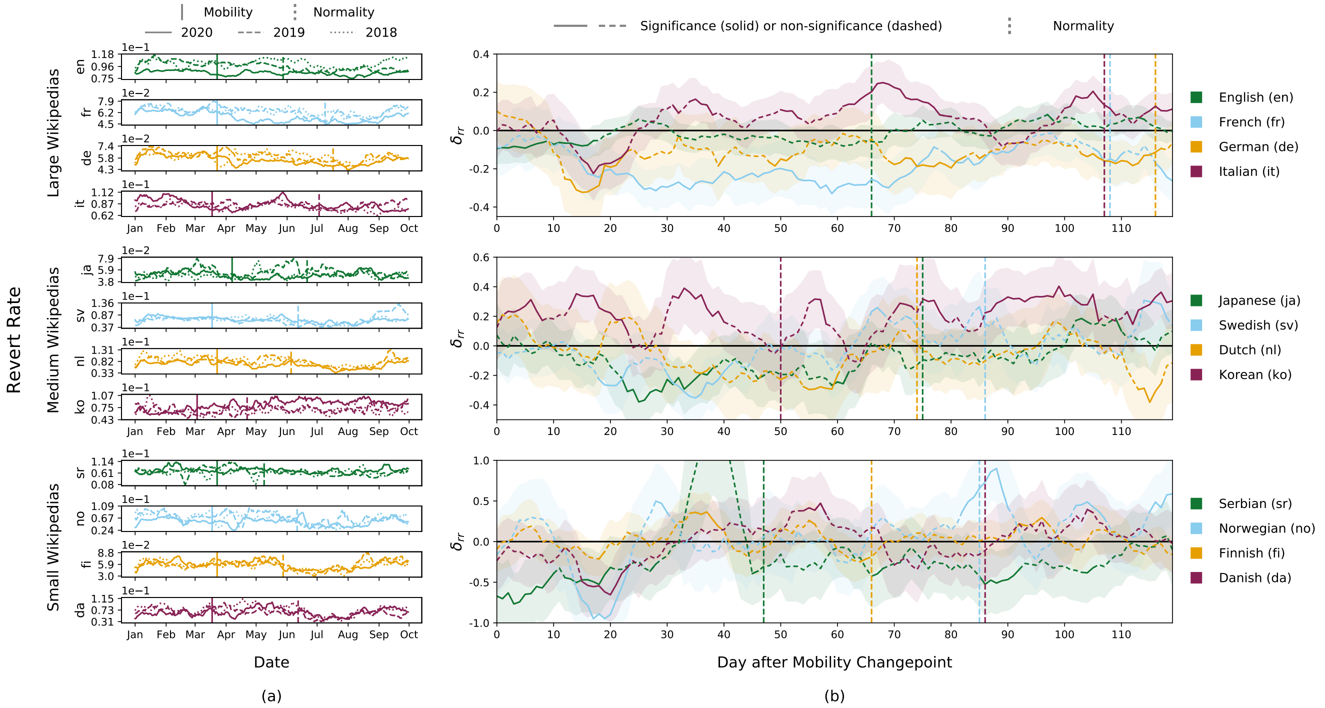

Supplementary Figure 19: **Revert rate during COVID-19 mobility restrictions (beginning 7 days later).** We vary our DiD for revert rate by moving the mobility (when restrictions take effect) and normality changepoint (when restrictions are lifted) to 7 days later. **a**, We show rolling seven-day average revert rate for 2018, 2019, and 2020 until October. **b**, We depict relative change in revert rate (rr) as retrieved from DiD via δ_{rr} (95% confidence interval as two standard deviations) and plot δ_{rr} for 120 left-aligned seven-day windows. We find no strong differences to the original experiment (Supplementary Fig. 6), as revert rates are relatively stable close to the mobility changepoint.
